# Supplementary material for: Stimuli-Responsive Track-Etched Membranes for Separation of Water–Oil Emulsions
Source: Membranes (Basel). 2023 May 17;13(5):523. doi: 10.3390/membranes13050523 (PMC10223353; doi:10.3390/membranes13050523)
Supplement: Supplementary file 1 [file membranes-13-00523-s001.zip › membranes-2332938-supplementary.pdf]

Supporting Materials

Stimuli-Responsive Track-etched Membranes for Separation of Water–Oil Emulsions

Indira B. Muslimova [1,2,*], Zhanna K. Zhatkanbayeva [1], Dias D. Omertasov [1], Galina B. Melnikova [1,3], Arman B. Yeszhanov [1,2], Olgun Güven [4], S.A.Chizhik [3], Maxim V. Zdorovets [1,2,5] and Ilya V. Korolkov [1,2,*]

[1] L.N. Gumilyov Eurasian National University, Satpaev str., 5, Astana, 010008, Kazakhstan
[2] The Institute of Nuclear Physics, Ibragimov str., 1, Almaty 050032, Kazakhstan
[3] A.V. Luikov Heat and Mass Transfer Institute of the National Academy of Sciences of Belarus, P. Brovki str., 15, 220072 Minsk, Belarus
[4] Department of Chemistry, Hacettepe University, Beytepe, Ankara 06800, Turkey
[5] Ural Federal University, Mira str. 19, 620002 Ekaterinburg, Russia
* Correspondence: bazarbaykyzy@list.ru (I.B.M.); i.korolkov@inp.kz (I.V.K) Tel.: +7-708-679-2137 (I.B.M.); +7-705-179-9083 (I.V.K.)

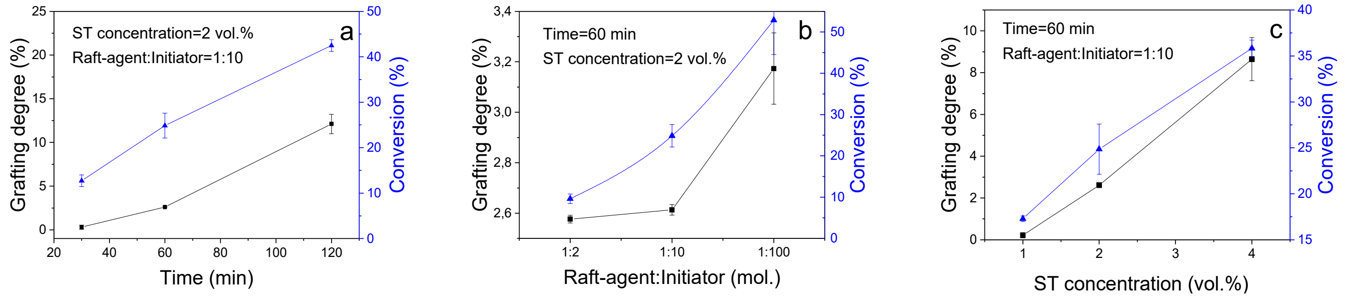

Figure S1. The effect of UV irradiation time (**a**), molar ratio of RAFT agent: initiator (**b**) and ST concentration (**c**) on the degree of grafting and conversion ST on PET TeMs (pore diameter of pristine PET TeMs is 2.0 ± 0.1 μm, pore density is 1 × 10^6 pore/cm^2, thickness is 23 μm).

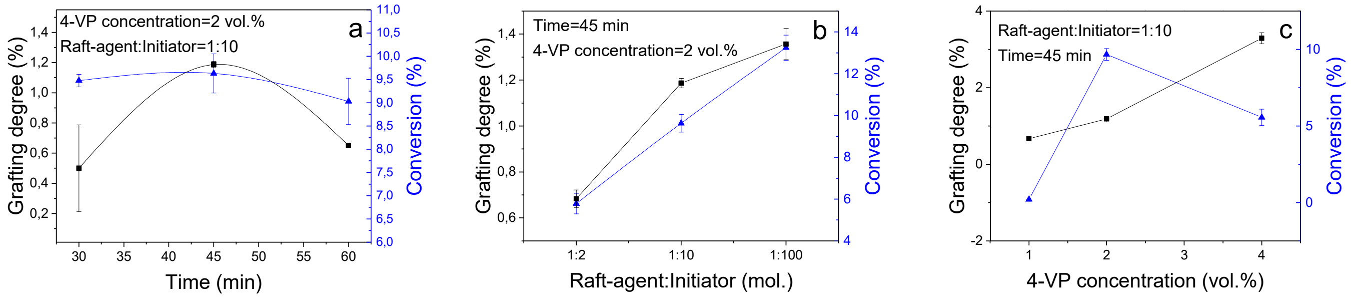

Figure S2. The effect of UV irradiation time (**a**), molar ratio of RAFT agent: initiator (**b**) and 4-VP concentration (**c**) on the degree of grafting and conversion 4-VP on PET TeMs (pore diameter of pristine PET TeMs is 2.0 ± 0.1 μm, pore density is 1 × 10^6 pore/cm^2, thickness is 23 μm).
